# Triazine Calixarene as a Dual-Channel Chemosensor for the Reversible Detection of Cu^2+^ and I^−^ Ions via Water Content Modulation

**DOI:** 10.3390/molecules30132815

**Published:** 2025-06-30

**Authors:** Fuyong Wu, Long Chen, Mei Yu, Liang Zhao, Lu Jiang, Tianzhu Shi, Ju Guo, Huayan Zheng, Ruixiao Wang, Mingrui Liao

**Affiliations:** 1 Department of Brewing Engineering, Moutai Institute, Renhuai 564500, China; chenlong@mtxy.edu.cn (L.C.); yumei@mtxy.edu.cn (M.Y.); zhaoliang@mtxy.edu.cn (L.Z.); jianglu@mtxy.edu.cn (L.J.); shitianzhu@mtxy.edu.cn (T.S.); guoju@mtxy.edu.cn (J.G.); zhenghuayan@mtxy.edu.cn (H.Z.); 2 Key Laboratory of Macrocycle and Supramolecular Chemistry of Guizhou Province, Guiyang 550025, China; ruixiao.wang@chambroad.com; 3 Biological Physics Laboratory, Department of Physics and Astronomy, School of Natural Science, The University of Manchester, Oxford Road, Manchester M13 9PL, UK

**Keywords:** fluorescent probe, dual-channel ion detection, Cu^2+^/I^−^ selectivity, reversible fluorescent response

## Abstract

Rationally designing and synthesizing chemosensors capable of simultaneously detecting both anions and cations via water content modulation is challenging. In this study, we synthesized and characterized a novel triazine calixarene derivative-based iodide and copper ion-selective fluorescent “turn-off” sensor. This dual-channeled fluorescent probe is able to recognize Cu^2+^ and I^−^ ions simultaneously in aqueous systems. The fluorescent sensor **s4** was synthesized by displacement reaction of acridine with 1, 3-bis (dichloro-mono-triazinoxy) benzene in acetonitrile. Mass spectrometry (MS), UV-vis, and fluorescence spectra were acquired to characterize the fluorescence response of **s4** to different cations and anions, while infrared (IR) spectroscopy and isothermal titration calorimetry (ITC) were employed to study the underlying selectivity mechanism of **s4** to Cu^2+^ and I^−^. In detail, **s4** displayed extremely high sensitivity to Cu^2+^ with over 80% fluorescence decrement caused by the paramagnetic nature of Cu^2+^ in the aqueous media. The reversible fluorescence response to Cu^2+^ and the responses to Cu^2+^ in the solution of other potential interferent cations, such as Li^+^, Na^+^, K^+^, Ca^2+^, Cd^2+^, Zn^2+^, Sr^2+^, Ni^2+^, Co^2+^ were also investigated. Probe **s4** also exhibited very good fluorescence selectivity to iodide ions under various anion (F^−^, Cl^−^, Br^−^, NO_3_^−^, HSO_4_^−^, ClO_4_^−^, PF_6_^−^, AcO^−^, H_2_PO_4_^−^) interferences. In addition to the fluorescent response to I^−^, **s4** showed a highly selective naked-eye-detectable color change from colorless to yellow with the other tested anions.

## 1. Introduction

Although some heavy metal ions are vital for maintaining human metabolism and play key roles in living systems, they can be highly toxic, posing significant environmental and health risks [1]. Iron (III), zinc (II), copper (II), cobalt (II), and manganese (II) are essential elements, yet their high concentrations can lead to adverse health effects [1,2,3]. Among these elements, copper (II) stands out for its critical involvement in numerous cellular processes, including gene expression and protein function [4]. Meanwhile, iodide is considered as an important ion among several other anions (PO_4_^3+^, HCO_3_^−^ and Cl^−^) for its various biological activities, for example, in thyroid function, normal growth, neurological activity, and brain function [5,6]. On the other hand, consumption of iodide in excess can cause adverse effects on human health [7].

Therefore, the development of increasingly selective and sensitive methods for the detection of iodide and copper is currently receiving considerable attention [4,8,9]. Several methods, including atomic absorption spectroscopy, inductively coupled plasma atomic emission spectrometry, electrochemical sensing, and the use of piezoelectric quartz crystals make it possible to detect low limits [10,11,12]. However, these methods require sophisticated equipment and are time-consuming, limiting their use to trained professionals. As an alternative, colorimetric sensors, which respond visually, offer several advantages, including simplicity, sensitivity, selectivity, and economic viability, without the need for specialized instrumentation [13,14].

Recently, Huang et al. synthesized a rhodamine-B derivative that functions as a “turn-on” probe for monitoring copper (II) ions in living cells [15]. This probe is colorless and exhibits weak fluorescence in acetonitrile. Upon the addition of copper (II) ions, however, the probe induces the appearance of a purple-red color and a pronounced orange-red fluorescence. The observed absorption and fluorescence changes are attributed to a two-step process. Initially, the copper (II) ions promote the ring opening of the probe. This is followed by a redox reaction between the probe and the copper (II) ions, leading to the reduction of copper (II) to copper (I). Lee and colleagues developed a novel iodide chemosensor that operated effectively over a wide concentration range of iodide ions in a CH_3_CN/H_2_O (99/1, *v*/*v*) solution, with no interference from other anions [16]. To enhance the detection limit of iodide in aqueous solutions, Chen et al. designed a new iodide ion chemosensor based on a hydrazone derivative. This sensor exhibited a distinct colorimetric response to iodide, achieving a detection limit as low as 1.0 × 10^−4^ mol/L through naked-eye color changes and 1.1 × 10^−6^ mol/L through changes in absorption spectra [17].

However, the development of a highly sensitive and selective chemosensor capable of simultaneously detecting multiple ions remains a significant challenge, particularly in response to specific conditions. Zhang et al. reported a fluorescent sensor with aggregation-induced emission (AIE) properties, which was highly sensitive to both iodide (I^−^) and mercury (Hg^2+^) ions. The sensor demonstrated its suitability for detecting low concentrations of I^−^ and Hg^2+^ in real samples [18]. Lee and co-workers developed sequential recognition of Zn^2+^ and Cu^2+^ using a new anthracene-containing dipyridylamine-based receptor [19]. This receptor exhibited highly selective and sensitive fluorescent “off–on” recognition of Zn^2+^, while the resulting receptor–Zn^2+^ complex displayed high selectivity to Cu^2+^ through a decrease in fluorescence intensity, demonstrating that the receptor–Zn^2+^ complex could detect Cu^2+^ via metal displacement. Qin et al. designed a multifunctional fluorescent chemosensor for detecting Cu^2+^ and Zn^2+^. The sensor exhibited significant fluorescence enhancement in the presence of Zn^2+^ but was quenched by Cu^2+^, an effect attributed to the paramagnetic nature of the Cu^2+^ species [20]. The development of a selective chemosensor for both I^−^ and Cu^2+^ ions is challenging due to the distinct properties of these ions, such as the large size and high polarizability of iodide and the paramagnetic nature of copper (II) [21,22]. In most of the reported Cu^2+^ fluorescent chemosensors, because of the paramagnetic nature of Cu^2+^, the binding of the metal ion causes a quenching of the fluorescence emission and leads to a “turn-off” signal [1]. However, very few Cu^2+^ chemosensors have the potential for I^−^ detection [23,24,25,26]. Patil and colleagues designed a PPT-1 receptor that showed a naked-eye-detectable color change from colorless to red in the presence of Cu^2+^ over the other tested cations [27]. In contrast, iodide ions did not change the color of PPT-1, but resulted a new spectroscopic response in the absorption spectrum at 232 nm, and a detection limit as low as 0.22 µM was achieved in aqueous solution. Meanwhile, rationally designing and synthesizing chemosensors capable of simultaneously detecting both anions and cations via water content modulation is still challenging.

1,3,5-Triazines are heterocyclic compounds containing three N atoms in a six-membered ring. These three nitrogen atoms can coordinate with metal ions, and their stability and properties are comparable to benzene due to π–electron conjugation. The compound exhibits high biological activity, including antibacterial, antihypertensive, and analgesic effects. Furthermore, the reactivity of the threew chlorine atoms on the triazine ring varies, allowing hierarchical substitution by controlling reaction conditions to selectively introduce one or more substituents on the triazine ring. This study utilized pyridine as the parent compound and 1,3,5-triazines as the connecting arm, to design and synthesize a triazine-based heteroaromatic compound **s4** for the highly sensitive and selective detection of both I^−^ ions and Cu^2+^ via water content modulation.

## 2. Results and Discussion

### 2.1. The Solvent Effects of Fluorescent Probe **s4**

**Figure 1:**
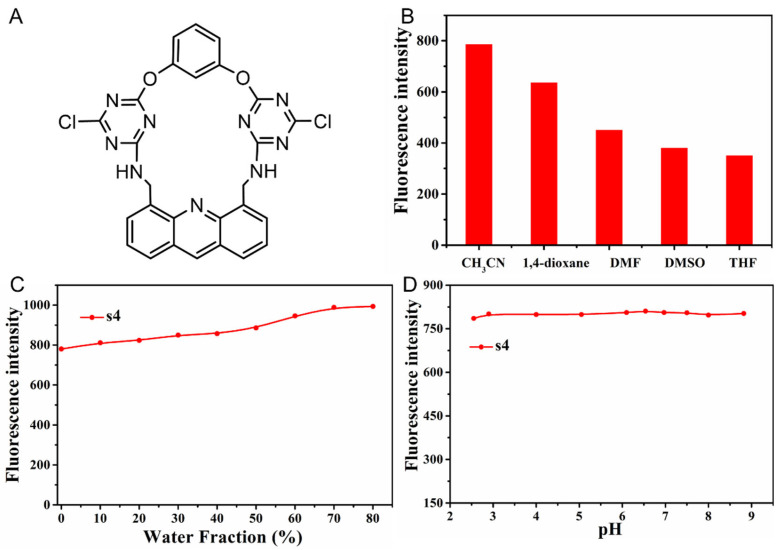
(**A**) Molecular structure of fluorescent probe **s4**. (**B**) The solvent effects on the fluorescent efficiency of **s4**; the test concentration of the compound was 10 µM. (**C**,**D**) The effects of H_2_O and pH on the fluorescent efficiency of fluorescent probe **s4** (10 µM), with fluorescent excitation and emission wavelength of λ_ex_/λ_em_ = 246/425 nm.

To study the solvent effects on the fluorescent efficiency of **s4**, the fluorescence spectra of probe **s4** were obtained in the presence of various solvents such as CH_3_CN, DMSO, DMF, 1,4-dioxane, and THF (Figure 1B). It was found that probe **s4** showed good solubility (100 µM **s4** in CH_3_CN for the further measurements) and the highest fluorescence emission intensity in CH_3_CN. Furthermore, the effects of water content and subsequent pH conditions in the solvent systems on the fluorescence intensity of **s4** were investigated (Figure 1C). When the water content ranged from 0 to 50%, the probe **s4** exhibited a slight increase in fluorescence intensity, and the fluorescence intensity increased further in the water content system > 50%. Based on the stable emission intensity of **s4** in the presence of the solvents with different water contents, the mixed solvent system CH_3_CN/H_2_O (*v*/*v*, 9/1) was selected for the following tests of pH effects (Figure 1D). The results indicated that probe **s4** showed relatively stable fluorescence emission in the tested range of pH from 2.5 to 8.8. All the subsequent experiments were carried out in presence of CH_3_CN/H_2_O (*v*/*v*, 9/1) with a pH at 6.5 to mimic physiological conditions.

### 2.2. The Fluorescent Emission Quenching of Probe **s4** in Response to Cu^2+^ and I^−^

**Figure 2:**
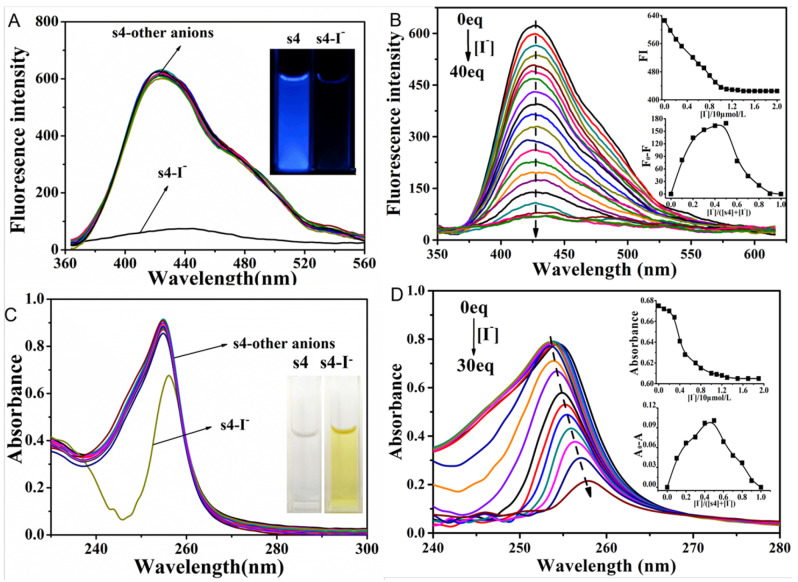
(**A**) Fluorescence and (**C**) UV–vis absorption spectra of different **s4**–anion complexes (the anions including F^−^, Cl^−^, Br^−^, I^−^, NO_3_^−^, HSO_4_^−^, ClO_4_^−^, PF_6_^−^, AcO^−^, H_2_PO_4_^−^), all the measurements were conducted with the concentration of **s4** at 10 μM. The inset graphs in (**A**,**C**) showed the color change of **s4** before and after addition with I^−^ under UV light and ambient light, respectively. (**B**) Fluorescence and (**D**) UV–vis absorption spectra of **s4**–I^−^ titration curves (I^−^ concentration ranging from 0 to 40 or 30 eq, CH_3_CN). Each fluorescence spectrum was recorded at 246 nm. The two insert graphs in (**B**,**D**) are the corresponding mole ratio (the upper part) and Job’s plot (the lower part) curves of **s4** reacted with I^−^, according to fluorescence (λ_ex_/λ_em_ = 246/425 nm) and UV–vis absorption spectra.

We evaluated the fluorescent selectivity of **s4** in response to various monovalent anions (F^−^, Cl^−^, Br^−^, I^−^, NO_3_^−^, HSO_4_^−^, ClO_4_^−^, PF_6_^−^, AcO^−^, H_2_PO_4_^−^) in CH_3_CN solution. As shown in Figure 2A, the fluorescence emission of probe **s4** was significantly quenched after the addition of I^−^ in comparison to the other anions. The corresponding inset graph showed the phenomenon that under 365 nm UV irradiation, the initial blue solution of probe **s4** became darker upon the addition of iodide ions. The results of the fluorescent titration curves showed the gradual fluorescence quenching of **s4** with the addition of I^−^ due to the heavy atom effect; the quenching efficiency reached 88% with the I^−^/**s4** ratio at 40 (Figure 2B). The observed static quenching effect was attributed to the spontaneous bonding of probe **s4** and I^−^ in the ground state. To verify the experimental results with the static quenching constant (*K_sv_*), which was calculated to be 3.09 × 10^4^ mol/L, the static fluorescence quenching rate constant (*K_q_*) was derived as 3.09 × 10^12^ mol/L, which was much greater than 2.0 × 10^10^ mol/L. The quenching trend suggested static quenching aligning with the formation of a **s4**–I^−^ complex. Furthermore, both the molar ratio and the Job method revealed the stoichiometric ratio of I^−^ to the probe **s4** as 1:1, with a detection limitation of 8.23 × 10^−8^ mol/L. In the UV–vis spectra of **s4** interacting with different anions, only I^−^ could lead to the decreased absorption intensity of the probe **s4** at 254 nm and cause the obvious red shift shown in Figure 2C. Moreover, the initial solution of the probe **s4** was colorless under white light, and it changes to a bright yellow color after the addition of iodide ions (inset graph in Figure 2C). With the increasing concentration of I^−^, the absorption intensity of **s4** at 254 nm decreased gradually along with a more obvious red shift. This indicated interactions between the molecules and iodide ions. Spectral titration of the probe **s4** with I^−^ suggested a 1:1 stoichiometric ratio of I^−^ to the probe **s4**, consistent with the previous fluorescence spectrum titration results. The stability constant of the I^−^ complex was calculated to be 4.36 × 10^4^ L·mol^−1^. Considering the UV spectrum, this red shift may have been due to the spontaneous bonding of probe **s4** and I^−^, which induced the formation of a complex between the molecule and iodide, thereby altering the original electronic configuration of the molecules, leading to the red shift in the spectrum. The transition from colorless to yellow under white light illumination suggests the involvement of heavy atom effects. In the excited state, the formation of an iodide anion–probe charge transfer complex, coupled with the reduction in energy due to the heavy atom effect, would have accounted for the observed fluorescence quenching [28,29]. However, the formation of **s4**–I^−^ complex did not change the molecular structure of **s4**. Meanwhile, the curves of fluorescent emission at high concentrations of iodine ion overlapped, indicating that the coordination of **s4** molecules with I^−^ aligned with the static quenching mechanism.

**Figure 3:**
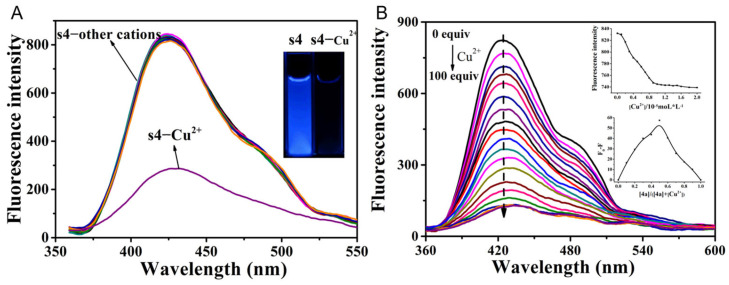
(**A**) The fluorescence spectra of different **s4**–cation systems (cations including Li^+^, Na^+^, K^+^, Ca^2+^, Cd^2+^, Zn^2+^, Sr^2+^, Ni^2+^, Co^2+^, Cu^2+^). All the measurements were conducted with the concentration of **s4** at 10 μM. The inset graph shows the color change of **s4** before and after addition of Cu^2+^ under UV light. (**B**) Fluorescence spectra of **s4**–Cu^2+^ titration curves (Cu^2+^ concentration ranging from 0 to 100 eq, CH_3_CN/H_2_O, 9/1, *v*/*v*, pH 6.5), at λ_ex_/λ_em_ = 246/425 nm. Each spectrum was recorded at 246 nm. The insert graphs in (**B**) show the mole ratio (the upper part) and Job’s plot (the lower part) of **s4**’s reaction with Cu^2+^.

In contrast, to improve the multifunctionality of the chemosensor, we simply added acidic aqueous solution into CH_3_CN to evaluate its cation recognition performance. Probe **s4** was further studied for its recognition of different cations. Various cations including Li^+^, Na^+^, K^+^, Ca^2+^, Cd^2+^, Zn^2+^, Sr^2+^, Ni^2+^, Co^2+^, and Cu^2+^ mixed with **s4** in CH_3_CN/H_2_O (*v*/*v*, 9/1, pH 6.5) were tested via fluorescence emission (Figure 3A). Among all the selected cations, the addition of Cu^2+^ significantly reduced the fluorescence intensity of the probe **s4**. The inset graph in Figure 3A shows the disappearance of blue fluorescence in **s4** solution, indicating the good detection capability of **s4** for Cu^2+^ in aqueous media. To describe the selectivity of **s4** to Cu^2+^ quantitatively, measurements of **s4** responses to Cu^2+^ with different molar ratios were carried out. The fluorescence quenching rate in **s4** reached 85% in the presence of a 100-fold concentration of Cu^2+^. Both the molar ratio and Job’s method indicated that the coordination ratio of probe **s4** to Cu^2+^ was 1:1. The binding constant of Cu^2+^ to probe **s4** was calculated to be 5.71 × 10^4^ mol/L. Although both I^−^ and Cu^2+^ caused fluorescence quenching, giving rise to the blue fluorescence disappearance in the solution, we also observed distinct colorimetric responses under white light: the solution turned from colorless to yellow upon addition of I^−^, whereas no color changing occurred with Cu^2+^ ion. This selective chromogenic behavior can be used as a dual-mode colorimetric detection chemosensor.

**Figure 4:**
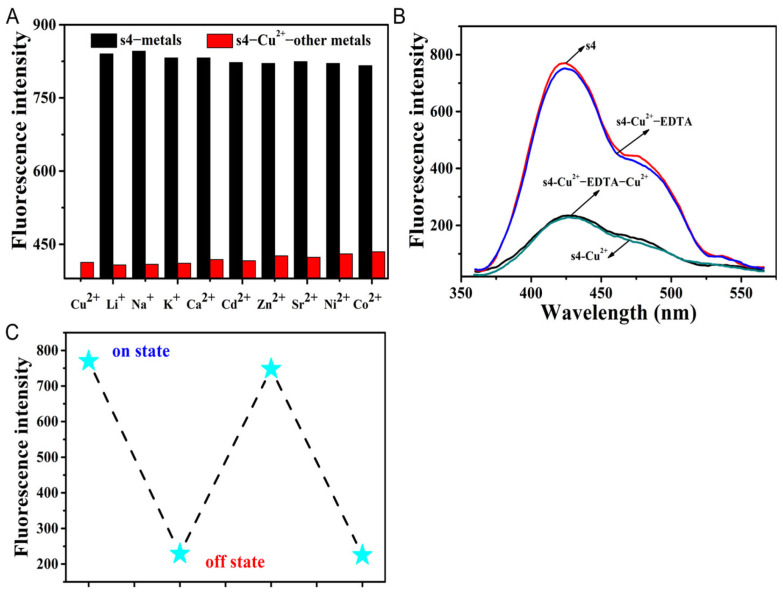
(**A**) The selective effects of Cu^2+^ complexing with **s4** in the solution environments of various metal ions (200 µM, Li^+^, Na^+^, K^+^, Ca^2+^, Cd^2+^, Zn^2+^, Sr^2+^, Ni^2+^, Co^2+^, Cu^2+^). The black bars represent the addition of the competing cations to the **s4** solution, while the red bars represent the addition of competing cations and Cu^2+^ to the **s4** solution. (**B**,**C**) The fluorescence spectra of reversible reaction between **s4** and Cu^2+^ upon addition of EDTA, and the corresponding change in fluorescent intensity between “on” and “off” states. The test concentration of **s4** in all the solution systems was 10 µM in CH_3_CN/H_2_O (9/1, *v*/*v*, pH 6.5), at a wavelength of λ_ex_/λ_em_ = 246/425 nm.

**Figure 5:**
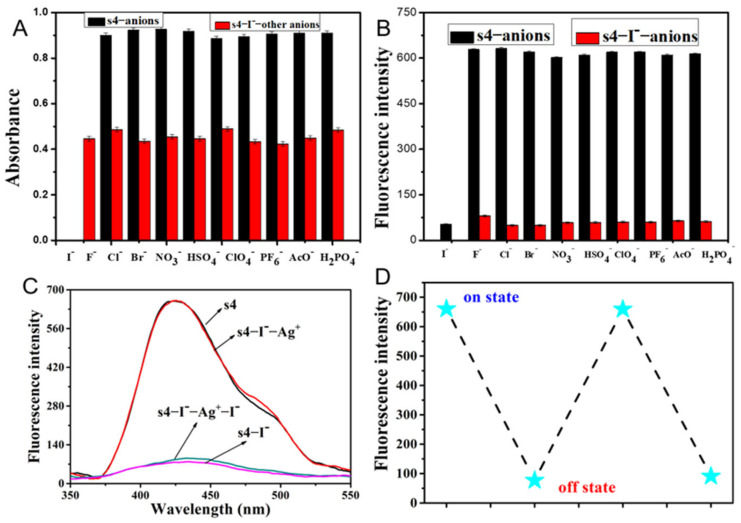
The selective effects of I^−^ complexing with **s4** in the solution environments of various anions (200 μM, F^−^, Cl^−^, Br^−^, I^−^, NO_3_^−^, HSO_4_^−^, ClO_4_^−^, PF_6_^−^, AcO^−^, H_2_PO_4_^−^), indicated by (**A**) UV–vis and (**B**) fluorescence spectra. The black bars represent the addition of the competing anions to the **s4** solution, while the red bars represent the addition of competing anions and I^−^ to the **s4** solution. (**C**) The fluorescence spectra of the reversible reaction between **s4** and I^−^ upon addition of Ag^+^, (**D**) and the corresponding change of fluorescent intensity between “on” and “off” states. The test concentration of **s4** in all the solution systems was 10 µM in CH_3_CN, at a wavelength of λ_ex_/λ_em_ = 246/425 nm.

To evaluate the selectivity of **s4**, the interference of coexisting metal ions or anions in the recognition of Cu^2+^ and I^−^, respectively, by the probe **s4** was investigated (Figure 4A and Figure 5B). The results of the fluorescent emission showed that **s4** exhibited high selectivity for both Cu^2+^ and I^−^, unperturbed by the addition of other competitive metal ions and anions. This was also quite evident from the iodide-induced shift in UV–vis absorbance maxima at 254 nm remaining unperturbed. These results confirm a great deal of selectivity for the detection of iodide ion compared with other anions (Figure 5A).

Reversibility is important for the continuous recyclability and reusability of chemosensors [10]. The reversible nature of **s4** towards Cu^2+^ in CH_3_CN/H_2_O (9/1, *v*/*v*, pH 6.5) with EDTA was characterized as shown in Figure 4B,C. Upon the addition of EDTA (20-fold molar concentration of probe **s4**) to the solution containing **s4** and Cu^2+^, the fluorescence intensity of the **s4**–Cu^2+^ complex recovery indicated the regeneration of **s4**. After that, the addition of 50-fold Cu^2+^ (relative to probe **s4**) resulted in a decrease in fluorescence emission at 425 nm as the Cu^2+^ was initially added. The results indicated that the excess Cu^2+^ reformed the **s4**–Cu^2+^ complex with free probe in solution, indicating that the coordination interaction was a reversible process. The time required for the complete reversible process was 10 s.

To confirm the reversible nature of **s4** towards I^−^, Ag^+^ was used as a competitive reagent (Figure 5C,D). With the addition of Ag^+^ (with a 20-fold molar ratio of the probe) to the probe **s4**–I^−^ complex solution, the fluorescence spectrum of the system was similar to that of the probe without adding I^−^, indicating that the addition of Ag^+^ had taken up the I^−^ from the complex, forming a AgI compound and releasing free probe. Subsequently, the same operation demonstrated that the system still exhibited good reversibility on fluorescence restoring, because the weak interactions between the probe **s4** and iodide allowed iodide ions to be easily displaced by the silver ions.

### 2.3. The Concentration and Temperature Effects of Fluorescent Probe **s4**

**Figure 6:**
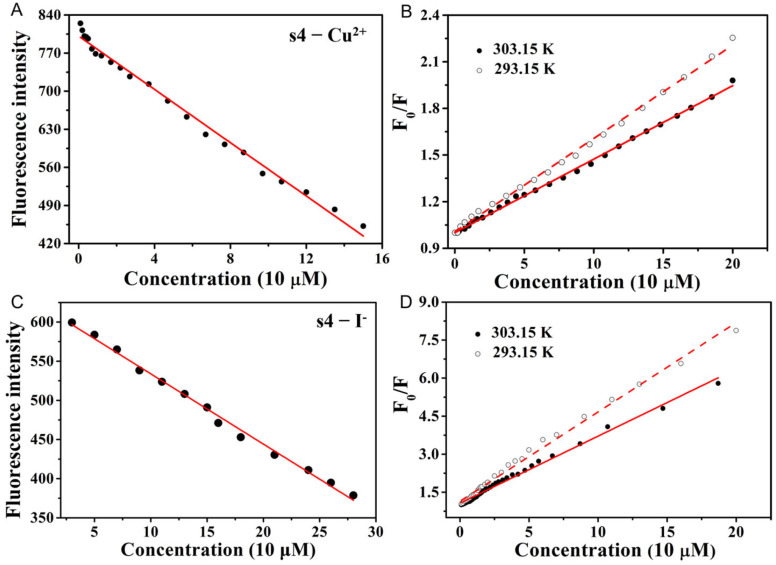
(**A**,**C**) Plots of fluorescence intensity at λ_ex_/λ_em_ = 246/425 nm for a mixture of **s4** (10 μM in CH_3_CN/H_2_O, *v*/*v*, 9/1, pH 6.5) with Cu^2+^ and I^−^ in CH3CN at different concentrations. (**B**,**D**) Stern–Volmer curves of **s4** with Cu^2+^ and I^−^ at temperatures of 293.15 and 303.15 K.

Under the experimental conditions described above, a calibration curve was established for determining the concentration of Cu^2+^ and I^−^, using fluorescence spectroscopy to analyze the probe **s4** (10 μM) (Figure 6A,C). The fluorescence intensity of the probe **s4** showed a linear correlation with the concentration of Cu^2+^ in the range from 8.0 × 10^−6^ mol/L to 3.5 × 10^−4^ mol/L, with a correlation coefficient R = 0.9978 (n = 11). For the probe **s4**–I^−^ calibration curve, there was a linear correlation in range of I^−^ concentration from 5.0 × 10^−6^ mol/L to 2.8 × 10^−4^ mol/L, with a correlation coefficient R = 0.9971 (n = 13). Ten sets of blank solutions were measured and the relative standard deviation (RSD) was calculated to be 2.31% (Cu^2+^) and 2.46% (I^−^), with a detection limit as low as 7.08 × 10^−8^ mol/L for Cu^2+^ ion and for 8.23 × 10^−8^ for I^−^ ion.

The effects of temperature on the fluorescence intensity of probe **s4** in the presence of different concentrations of Cu^2+^ or I^−^ were measured, and the fitting curves were obtained according to the previously published method, as shown in Figure 6B,D [17,21,30]. The slopes of the quenching curves for both probe **s4**–Cu^2+^ and **s4**–I^−^ systems decreased with the temperature increase from 293.15 K to 303.15 K, indicating a static quenching led by temperature effect. The following Stern-Volmer equation was applied:

F_0_/F = 1 + *K_sv_* [Q] = 1 + *K_q_* × *τ_o_* [Q]

The static quenching constant *K_sv_* was calculated to be 5.97 × 10^3^ mol/L (Cu^2+^) and 3.09 × 10^4^ mol/L (I^−^), and the static fluorescence quenching rate constant *K_q_* was obtained to be 5.97 × 10^11^ (Cu^2+^) and 3.09 × 10^12^ (I^−^). The *K_q_* values for Cu^2+^ and I^−^ were both greater than 2.0 × 10^10^ L·mol^−1^, indicating that the quenching process was caused by the formation of **s4**–Cu^2+^ and **s4**–I^−^ complexes, respectively.

### 2.4. The Mechanism of Cu^2+^ Fluorescent Probe **s4**

**Figure 7:**
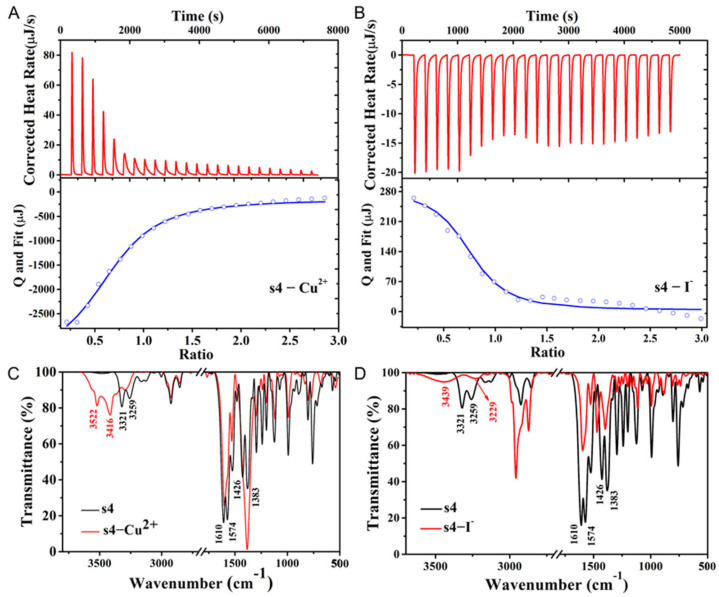
(**A**,**B**) Binding of **s4** to Cu^2+^ or I^−^. Isothermal calorimetric titration (ICT) of **s4** with Cu^2+^ in DMF and I^−^ in CH_3_CN (upper part). Raw experimental data and calorimetric titration curves for the binding of Cu^2+^ or I^−^ to **s4** (lower part). (**C**,**D**) IR spectra of compound **s4** and **s4**–Cu^2+^ or **s4**–I^−^ in KBr disks.

To explore the underlying mechanisms between **s4** and Cu^2+^ or I^−^ for the emission quenching of **s4**, the corresponding ITC measurements in CH_3_CN solvent were carried out (Figure 7A,B). The binding of probe **s4** to Cu^2+^ is an endothermic reaction and tends to be equilibrate with the increased Cu^2+^ concentration. From the nonlinear fitting curve of the molar ratio of probe **s4** to Cu^2+^ based on the reaction heat, the binding constant was calculated to be *K_a_* = (8.55 ± 0.52) × 10^4^ mol/L, number of binding sites n = 0.964 ± 0.030, molar binding enthalpy ΔH° = (−331.2 ± 2.04) kJ/mol, molar binding entropy TΔS° = −302.92 kJ/mol, and molar binding free energy ΔG° = (−28.28 ± 2.04) kJ/mol. The coordination ratio and binding constant were in agreement with the spectral measurement results. Unlike the binding between **s4** and Cu^2+^, the ITC curve of the interaction between probe **s4** and I^−^ in CH_3_CN solvent reflected a typically exothermic reaction. Through the non-linear fitting of binding reaction heat between the probe **s4** and I^−^, the parameters that were yielded included binding constant *K_a_* = (7.20 ± 0.63) × 10^4^ mol/L, number of binding sites n = 0.921 ± 0.055, molar binding enthalpy ΔH° = (9.35 ± 1.19) kJ/mol, molar binding entropy TΔS° = 37.09 kJ/mol, and molar binding free energy ΔG° = (−27.74 ± 1.19) kJ/mol. These results indicate that the binding of the probe **s4** to iodide ion is a spontaneous procedure. The coordination ratio and binding constant were consistent with the results obtained from the fluorescence spectra.

To explore the sensing mechanism of **s4** to Cu^2+^ and I^−^ on the molecular level, the IR spectra were further investigated, which revealed characteristic structural changes of **s4** occurring upon interaction with different ions (Figure 7C,D). In the IR spectra of **s4** before and after addition with Cu^2+^, the N-H stretching vibrations of the probe **s4** at 3259 cm^−1^ and 3321 cm^−1^ clearly shifted towards higher wavenumbers at 3416 cm^−1^ and 3522 cm^−1^. Additionally, the double peaks of the C-N in **s4** (at 1383 cm^−1^ and 1426 cm^−1^) merged into a single peak with increased intensity after **s4** binding with Cu^2+^. The peaks of the N-H bending vibrations at 1574 cm^−1^ and 1610 cm^−1^ became single peaks with reduced intensity, suggesting that Cu^2+^ may participate in coordination with the N atom in the secondary amine of the probe **s4**. Similarly, when **s4** coordinated with I^−^, the stretching vibration absorption peaks of N-H shifted to higher wavenumbers along with the peak becoming broader. Moreover, the C-N peaks at 1383 cm^−1^ and 1426 cm^−1^ shifted to higher wavenumbers at 1398 cm^−1^ and 1471 cm^−1^, and their intensities decreased. The N-H bending vibrations at 1574 cm^−1^ and 1610 cm^−1^ became single peaks with weakened intensities. These observations suggest that I^−^ may participate in the coordination process with the secondary amine in the probe molecule.

## 3. Conclusions

In summary, we have developed a novel chemosensor **s4**, featuring a triazine calixarene structure. This sensor exhibits specific selectivity and high sensitivity in detecting copper (II) ions (Cu^2+^) and iodide ions (I^−^) via water content modulation. The fluorescent response to Cu^2+^ is marked by a striking color transition from blue to dark under UV light, which aligns with the paramagnetic properties of Cu^2+^. The remarkable colorimetric sensing of probe **s4** confirmed a 1:1 (probe **s4**–Cu^2+^) binding model with the detection limit down to 7.08 × 10^−8^ mol·L^−1^. Other than the fluorescent recognition of I^−^, a unique colorimetric response to I^−^ based on UV-vis spectra is realized through the coordination with probe **s4**. In particular, competitive anions such as F^−^, Cl^−^, Br^−^, NO_3_^−^, HSO_4_^−^, ClO_4_^−^, PF_6_^−^, AcO^−^, H_2_PO_4_^−^ did not afford any obvious interference response. The detection limits of I^−^ were found to be 8.23 × 10^−8^ mol·L^−1^ according to the naked-eye color changes and absorption spectral changes respectively. This recognition behavior makes probe **s4** a potential tool to detect Cu^2+^ and I^−^ in environmental and life sciences.

## 4. Materials and Experimental Methods

All the chemicals were purchased commercially as analytical pure reagents. Triazine calixarene was purchased from Sigma (Sigma Aldrich Technology., Ltd., St. Louis, MO, USA) and used without further purification. The metal ion solution was prepared from its perchlorate. The anion solution was prepared using its tetrabutylammonium salt with acetonitrile. High-purity water (ElgaUltrapure with a resistivity of 18.2 MΩ·cm) was used in all the experiments.

Mass spectra were recorded on an Agilent LC/MSD spectrometer, based on infusion methods. UV–vis absorption studies were carried out on a TU-1901 UV–Vis spectrophotometer (Beijing Puxi General Instrument Co., Ltd., Beijing, China). Fluorescence measurements were performed on a Varian Cary Eclipse spectrofluorimeter equipped with quartz cuvettes of 1 cm path length. The excitation and emission slit widths were 5.0 nm. All absorption and emission spectra were recorded at 24 ± 1 °C. Stock solutions of probe **s4** (100 µM in CH_3_CN/H_2_O, 9:1, *v*/*v*, pH 6.5) were prepared immediately before the experiments. The solutions of metal ions were prepared from perchlorates of Li^+^, Na^+^, K^+^, Ca^2+^, Cd^2+^, Zn^2+^, Sr^2+^, Ni^2+^, Co^2+^. The solutions of anions were prepared from tetrabutylammonium salts of F^−^, Cl^−^, Br^−^, I^−^, NO_3_^−^, HSO_4_^−^, ClO_4_^−^, PF_6_^−^, AcO^−^, H_2_PO_4_^−^. Stock solutions of Cu^2+^ and I^−^ was prepared with Cu(ClO_4_)_2_·6H_2_O and tetrabutylammonium iodide at a concentration of 2 mM. Tris–HCl solution with a concentration of 2 mM was prepared as buffer solution to use for pH control of the solvent systems.

### 4.1. Molecular Design and Synthesis

Based on the previous work using calixarene as the parent body, a new macrocycle molecule was synthesized, in which acridine as a main body covalently linked with the triazine derivative group. The above reagents (acridine and triazine derivative) were synthesized according to procedures published previously [26,31]. The synthesis method followed the following procedure. N,N-Diisopropyl-ethylamin (DIPEA, 10.5 mM in acetonitrile) as an acid binding agent was mixed with aminomethyl acridine (0.42 mM in acetonitrile); after that, the triazine derivative (0.42 mM 1, 3-bis (dichloro-mono-triazinoxy) benzene in acetonitrile) was dropped into the solution. The final macrocyclic calixarene was obtained by the nucleophilic substitution reaction in a nitrogen atmosphere under 40 °C for 48 h. The specific synthesis and characterization are described in a previous report [32].

### 4.2. Fluorescence and UV-Vis Spectra

In a series of 10.0 mL volumetric flasks, 1.0 mL of probe **s4** acetonitrile stock solution, 1.0 mL Tris-HCl buffer, and 1.0 mL of Cu^2+^ or other metal ion (I^−^ or other anion solution in case of anion recognization) aqueous solution were added, and diluted to the mark with CH_3_CN/H_2_O (*v*/*v*, 9/1) for further use in UV–vis and fluorescence spectral analysis. The excitation wavelength of the fluorescence spectra was set at 246 nm.

### 4.3. Isothermal Titration Calorimetry

The ITC measurements were carried out with Nano ITC (TA Instrument Co., Ltd., USA). Firstly, 1.0 mL probe **s4** solution (100 µM in DMF) was injected into the calorimetric titration cell. The titration syringe was filled with 250 µL Cu^2+^ solution (1 mM in DMF). Each 6 µL Cu^2+^ solution was injected into the calorimetric cell at an interval of 180 s, while keeping stirring at a rate of 250 rpm until equilibrium. The I^−^ titration procedure was the same as above, with the solvent selected as CH_3_CN. All the tests were carried out at a temperature of 298.15 K.

### 4.4. IR Spectra

The IR spectra for the chemosensor **s4** and the **s4**–I^−^ and **s4**–Cu^2+^ complexes were recorded. The IR spectroscopic analysis was conducted by using a Bruker Vertex 70 FTIR Spectrophotometer (Bruker, Billerica, MA, USA). A uniform resolution of 2 cm^−1^ was maintained in all cases.

## Data Availability

Data are contained within the article.
